# Postoperative symptomatic vasospasm in pituitary surgery: case series and systematic review – a matter of blood?

**DOI:** 10.1007/s10143-026-04182-4

**Published:** 2026-02-19

**Authors:** Lennart W. Sannwald, Gregor Durner, Hans-Georg M. Kesseler, Nina Kreße, Michal Hlavac, Andreas Knoll, Johannes Roßkopf, Ralph W. König, Christian R. Wirtz, Andrej Pala

**Affiliations:** 1 Department of Neurosurgery, Ulm University, Ulm, Germany; 2 Department of Neuroradiology, Ulm Unversity, Ulm, Germany

**Keywords:** Pituitary surgery, Transsphenoidal surgery, Transcranial surgery, Vasospasm, Delayed cerebral ischemia, Tumor bed hemorrhage

## Abstract

Vasospasm is an unusual complication after pituitary surgery with potentially severe sequelae. There is limited information available in the literature concerning risk, patterns, outcomes and therapy. This systematic review investigates reported cases between 1956 and 2025 to improve assessment and management of this phenomenon. Pubmed database was systematically screened between 1st January 1969 and 10th October 2025 for medical subject headings (MeSH-terms) ‘Vasospasm’ OR ‘Delayed cerebral ischemia’ OR ‘Delayed neurological deficit’ AND ‘pituitary surgery’. The first description by Krayenbühl in 1956 was included as only report that could be identified before 1969. Overall, 262 articles were identified and abstracts screened. Afterwards, 59 articles were reviewed identifying 114 patients and excluding 205 patients. Additionally, three original cases are reported. Vasospasm after pituitary surgery is most often reported after resection of large non-functioning pituitary adenomas (50/117) or craniopharyngiomas (25/117). Vasospasm manifests predominantly between the fifth and twelfth postoperative day (73%) with altered sensorium, palsy or aphasia and affects ICA, MCA, ACA or their combinations in more than 90% of cases. Tumor bed hemorrhage was present in 75% of cases with reported early postoperative MR or CT imaging. Therapy is not standardized, more than half of reported patients suffer permanent neurological morbidity (36.7%) or mortality (19.4%). Vasospasm after pituitary surgery is rare but devastating in an otherwise benign disease. This systematic review suggests an association with tumor bed hemorrhage and marked similarities with subarachnoid hemorrhage induced vasospasm. Patients at risk should be defined preoperatively and closely monitored postoperatively.

## Introduction

Vascular spasm is an unusual complication after pituitary surgery with potentially severe sequelae. There is limited information available in the literature concerning risk, patterns, outcomes and optimal therapy. This review investigates reported cases between 1956 and 2025 to improve assessment and management of this phenomenon.

The wall structure of subarachnoid arterial vessels varies significantly from those in the rest of the body with an overall thinner wall but a proportionately higher ratio of muscular tunica media. This has been hypothesized to predispose those arteries for vascular spasm [44]. Since the seminal description of angiographic vasospasm in association with subarachnoid hemorrhage by Eckert and Riemenschneider, vasospasm has increasingly been considered as pathophysiological origin of delayed cerebral ischemia and neurological deterioration [23, 46]. However, only severe angiographic vasospasm is associated with neurological worsening [25]. Prevention of surgically and mechanically induced vasospasm with papaverine during intraoperative dissection has been focused on since the beginnings of microneurosurgery [79]. Delayed vasospasm after disease ictus has classically been associated with vasoconstrictive effects of subarachnoid blood clots - potentially as protective mechanism to endogenously close arterial rupture sites, although the peak incidence after subarachnoid hemorrhage is classically reported to be between the 6th and 8th day after bleeding [26, 73, 79].

Risk and severity of vasospasm are associated with amount of subarachnoid blood clots and not the extent of intraventricular or intraparenchymatous hemorrhage. Extensive blood clot removal mitigates arterial vasospasm [22, 26, 54, 55, 63]. Additionally, vasospasm after subarachnoid hemorrhage preferentially affects vessels of the anterior circulation with a potential predilection for the anterior cerebral artery more than the middle cerebral artery [26]. As only severe cerebral vasospasm has been linked to ischemia or delayed neurological deficit following subarachnoid hemorrhage, research interest has shifted toward additional mechanisms of ischemia such as microthrombosis, neuroinflammation, and cortical spreading depolarization. The term ‘vasospasm’ is now being replaced by ‘delayed cerebral ischemia’ to more accurately represent the range of potential pathophysiological processes in subarachnoid hemorrhage [32, 47].

Early on hypothalamic damage was associated with risk of severe vasospasm since it can be initiated by subarachnoid injection of hypothalamic tissue serum but not by injection of hypothalamic hormones oxytocin and vasopressin alone [74, 76]. The combination of extensive direct and indirect (mass shift by rapid resection of large benign and slow growing tumor masses) surgical manipulation of perisellar arteries, localized postoperative blood clots and potential reversible or irreversible damage to the hypothalamic-pituitary-axis put surgery of large pituitary masses at particular risk of postoperative vasospasm.

The first description of postoperative vasospasm after subfrontal resection of a pituitary tumor by Krayenbühl in 1956 and after transsphenoidal tumor resection by Camp et al. in 1980 marked the beginning of an increasing interest in postoperative vasospasm in pituitary surgery [15, 43]. Following our analysis of three own cases and recognizing the limited availability of comprehensive data on vasospasm as a complication of pituitary surgery, the literature since the first description of this phenomenon in 1956 was systematically reviewed for reports of postoperative vasospasm associated with all types of pituitary surgery. The results of this analysis of clinical characteristics and outcomes are reported.

## Methods

### Case reports

Three original cases identified by the local registry for sella pathologies of the neurosurgical department of Ulm University at the district hospital of Günzburg, a neurosurgical tertiary care center in Bavaria, southern Germany, were reviewed regarding parameters described below in the literature review section. This study was performed in line with the principles of the Declaration of Helsinki. Scientific analysis and publication of cases from the registry were approved by the local ethics committee of Ulm University (identification number 225/24).

### Systematic review

**Fig. 1:**
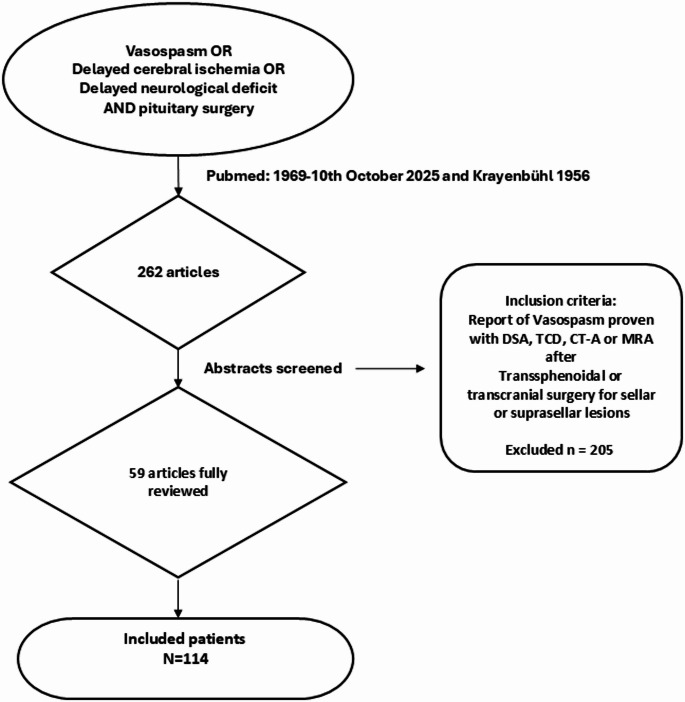
Review Process. Pubmed was systematically screened between 1st January 1969 and 10th October 2025 for medical subject headings (MeSH-terms) ‘Vasospasm’ OR ‘Delayed cerebral ischemia’ OR ‘Delayed neurological deficit’ AND ‘pituitary surgery’. Additionally, the first description by Krayenbühl in 1956 was included. Overall, 262 articles were identified and abstracts screened. Afterwards, 59 articles were reviewed fully identifying 114 patients and excluding 205 reported patients

To screen the PubMed-database for reports of postoperative vasospasm following pituitary surgery, regardless of approach or lesion type, the medical subject headings (MeSH-terms) ‘(((Vasospasm) OR (Delayed cerebral ischemia) OR (Delayed neurological deficit)) AND (pituitary surgery))’ were used (see Fig. 1). Postoperative vasospasm was defined as postoperative unexplained immediate or delayed neurological deficit or ischemia in combination with detection of cerebral vasospasm in related cerebral arteries by digital subtraction angiography, CT or MR angiography or transcranial doppler sonography. Thus, all cases included in this review were diagnosed with vasospasm either by transcranial doppler sonography, CT- or MR-angiography or digital subtraction angiography. Because postoperative vessel imaging is not routinely used after pituitary surgery, detection of postoperative vasospasm was considered to be mainly related to unexpected postoperative deficits.

Literature was systematically screened by the first author from 1st January 1969 to 10th October 2025 (only the report by Krayenbühl in 1956 was identified before 1969 by screening bibliographies of all reports after 1969). Additionally, citations of reviews, case series and case reports that were identified by the MeSH-terms mentioned above were screened for additional cases. Consequently, 262 articles were identified (including Krayenbühl 1956) and their abstracts screened for reports of postoperative vasospasm after pituitary surgery to assess eligibility. Cases with pituitary apoplexy and related preoperative vasospasm were excluded. Fifty-nine articles were included and fully reviewed. Number of cases, age, sex, pathological entity and its extension, surgical approach, association with recurring lesion and pituitary apoplexy, day of symptom onset, type of neurological deficit, outcome with neurological morbidity or mortality at last follow up, infarction in imaging, postoperative electrolyte disturbance, pituitary insufficiency, CSF leakage and meningitis as well as therapeutic measures were recorded if reported. If vasospasm was recorded as part of a case series where the parameters mentioned above could not be tracked to individual cases and were reported in summary only, the report was included but is depicted in the results section separately. Two cases reported by Tsunoda in 1972 and cited by Aoki et al. were not included due to lack of access to the original publication text [7]. Because of inconsistent data reporting across various case reports the number of cases in which each variable reported in this review was not specified is also reported in this review.

Analysis was restricted to descriptive statistics due to the heavily biased nature and often incompletely reported characteristics of retrospectively collected case reports and series. Incomplete data is explicitly stated regarding every outcome measure. Tables and diagrams were created with Microsoft Excel 2007. After a brief report of three original cases the results of the literature review and the original cases are presented together. This systematic review adheres to the PRISMA 2020 guidelines where applicable due to limitations of descriptive statistics of case reports and small case series. This review is not registered.

This is the most comprehensive review to date regarding postoperative vasospasm after pituitary surgery and aims at providing an overview of clinical presentation and characteristics of cases of postoperative vasospasm in pituitary surgery as reported in world literature.

## Results

### Case 1

**Fig. 2:**
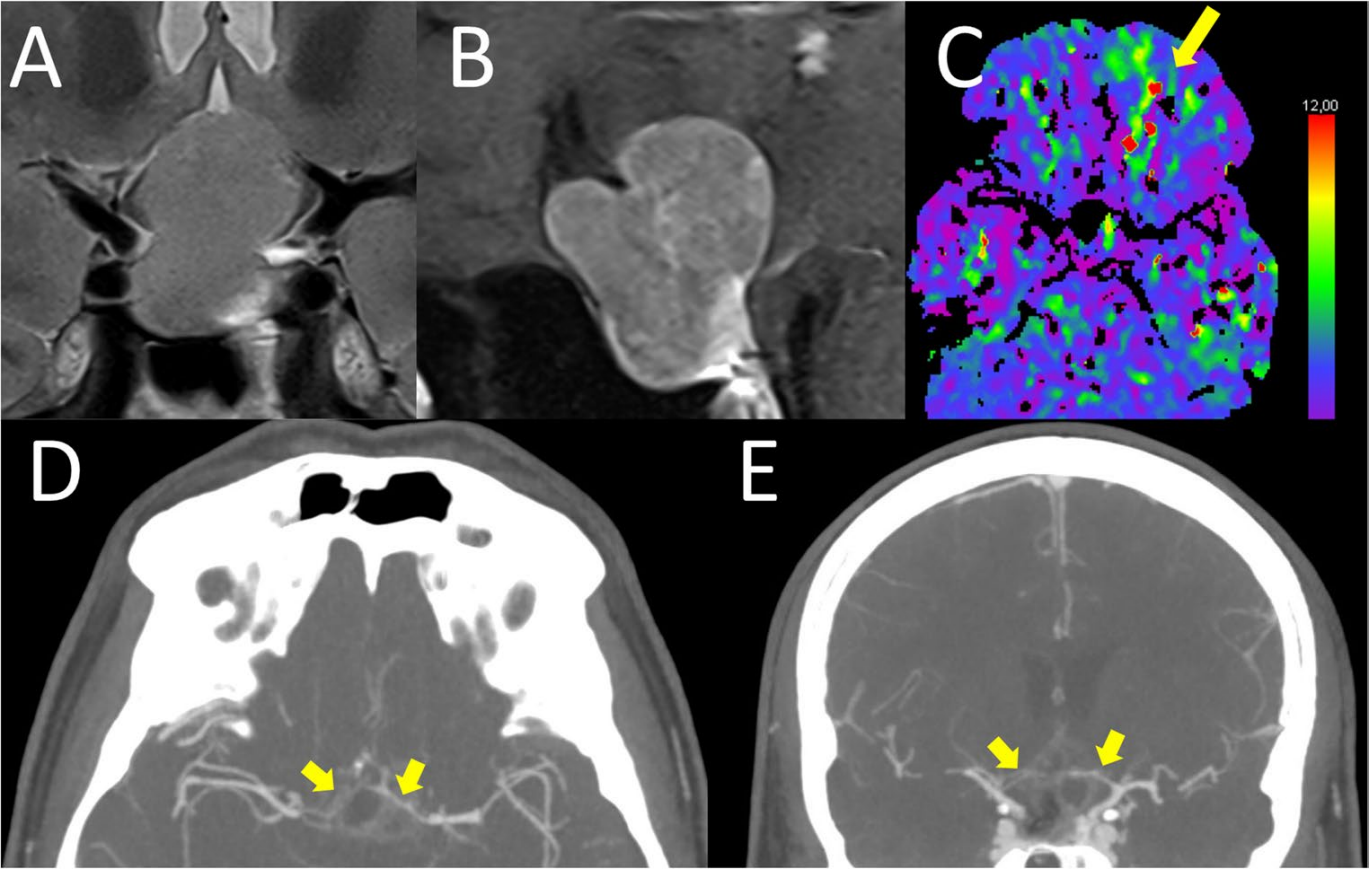
Case 1–51 year old male with lactotroph pituitary neuroendocrine tumor. **A** : preoperative T2-weighted MR coronal section showing a sellar tumor with suprasellar extension elevating the floor of the third ventricle. **B**: preoperative sagittal T1-weighted contrast-enhanced MR section demonstrating two suprasellar tumor extensions above the tuberculum sella towards the anterior cerebral arteries as well as towards the third ventricular floor. **C**: Postoperative day 12, Tmax map of perfusion CT, arrow pointing out spots of delayed perfusion in the left orbitofrontal area supplied by the anterior cerebral artery. **D** (axial) & **E** (coronal): contrast-enhanced CT angiography on postoperative day 12, arrows marking extreme vessel narrowing of A1 segment of both anterior cerebral arteries indicative of vasospasm

A 51 year old male (Fig. 2) was diagnosed with a sellar and suprasellar lesion due to increasing visual field defects. The tumor showed extension around the complex of the anterior communicating artery as well as A1 and A2 segments of the anterior cerebral artery on both sides and beginning infiltration of the right cavernous sinus. Laboratory tests revealed no preoperative pituitary insufficiency and hyperprolactinemia (386 ng/ml, normal range 3.8–21.2). Antiplatelet medication (ASA and prasugrel due to heart disease) was stopped a week preoperatively. Intraoperatively, after an endoscopic transsphenoidal transsellar and transtubercular approach the tumor was well delineated and could be dissected from the remaining pituitary gland. Due to strong adhesions to the A2 segment a remnant of tumor was left before reconstruction of the skull base with fat and synthetic dural substitute. Postoperatively, the patient developed transient arginine vasopressin deficiency but otherwise showed an uneventful recovery under substitution with oral hydrocortisone. Pathological examination demonstrated a lactotroph pituitary neuroendocrine tumor. Due to his good clinical condition the patient received no routine postoperative imaging to detect tumor bed hemorrhage. On the 12th postoperative day the patient presented to the emergency department with headache and a fluctuating palsy of the left leg (Medical research council grade (MRC) 3/5). Emergent imaging with CT-angiography and -perfusion showed corresponding vasospasm of both anterior cerebral arteries and a perfusion deficit. However, after spontaneous improvement in the emergency department the patient left against medical advice and could not be contacted for follow up.

### Case 2

**Fig. 3:**
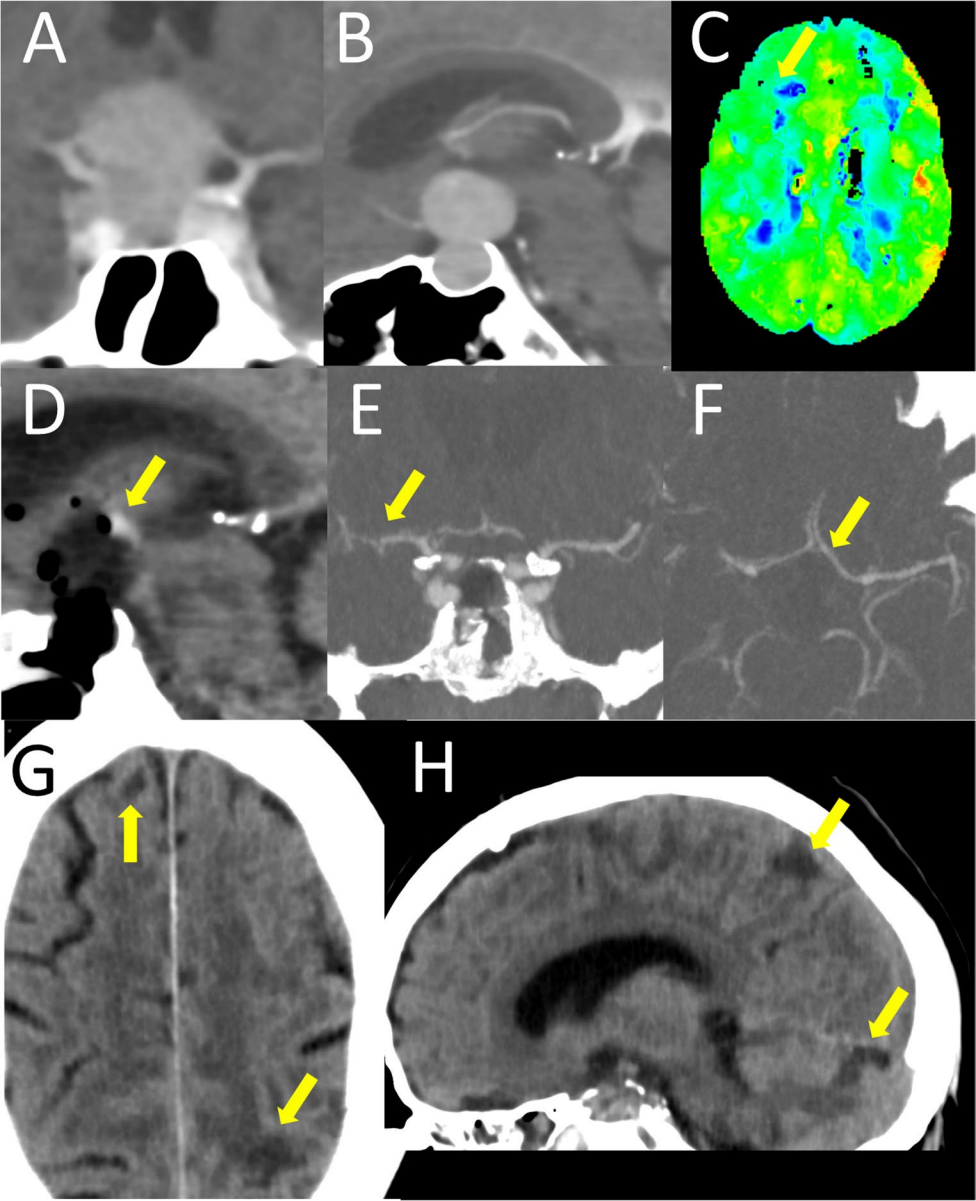
Case 2–54 year old male with non-functioning pituitary neuroendocrine tumor. **A** (coronal) & **B** (sagittal): contrast-enhanced cranial CT imaging showing a sellar lesion with suprasellar extension and snowman-like configuration with contact to both A1 segments of the anterior cerebral arteries. **C**: Postoperative day 5, Tmax map of perfusion CT, blue color indicates delayed perfusion whereas green, yellow and red areas indicate rapid perfusion, arrow pointing out delayed perfusion in the watershed zone of the anterior and middle cerebral artery. **D**: Postoperative representative sagittal CT section with arrow pointing out focal postoperative tumor bed hemorrhage. **E** (coronal) & **F** (sagittal): contrast-enhanced CT angiography on postoperative day 5 with arrows indicating reduced caliber of both A1 segments of the anterior cerebral arteries and right M1 segment of the middle cerebral artery. **G** (axial) & **H** (sagittal): cranial CT without contrast agent, arrows demonstrating lacunar ischemic defects in the watershed area of the anterior cerebral artery as well as a single cerebellar defect

A 54 year old male (Fig. 3) developed progressive loss of vision over several weeks which worsened acutely at the day of presentation to almost complete blindness on the right eye. Due to an implantable cardioverter defibrillator the patient received only CT imaging demonstrating a sellar und suprasellar mass with a snow-man-like configuration. Both the floor of the third ventricle and the proximal anterior cerebral arteries were stretched around the tumor. The patient was transferred to the OR for emergent endoscopic transsphenoidal resection of the lesion. Intraoperatively, the soft adenoma tissue was removed completely after gradual dissection from adhesions to the opticchiasma and floor of the third ventricle as well as hemostasis of relevant bleeding from the cavernous sinus. The sellar floor was reconstructed with abdominal fat. Histopathology showed non-functioning pituitary neuroendocrine tumor. Postoperatively, the patient reported improved vision and was substituted with hydrocortisone. He presented increasingly disoriented and on day five developed foot drop on both sides (MRC 2/5), numbness of both calves and urinary retention. Emergent imaging with CT-angiography and -perfusion showed vasospastic anterior cerebral arteries with associated perfusion deficit, already demarcated infarcts on both sides (as well as the cerebellum) and mild tumor bed hemorrhage at the floor of the third ventricle. Therapy was initiated with intravenous nimodipine and hemodynamic augmentation, after which the patient stabilized clinically. Four months later after rehabilitation, the patient still suffered from complete pituitary insufficiency and arginine vasopressin deficiency. Both vision and foot drop (MRC 4/5) had improved significantly, while the patient needed a urinary catheter due to neurogenic voiding dysfunction.

### Case 3

**Fig. 4:**
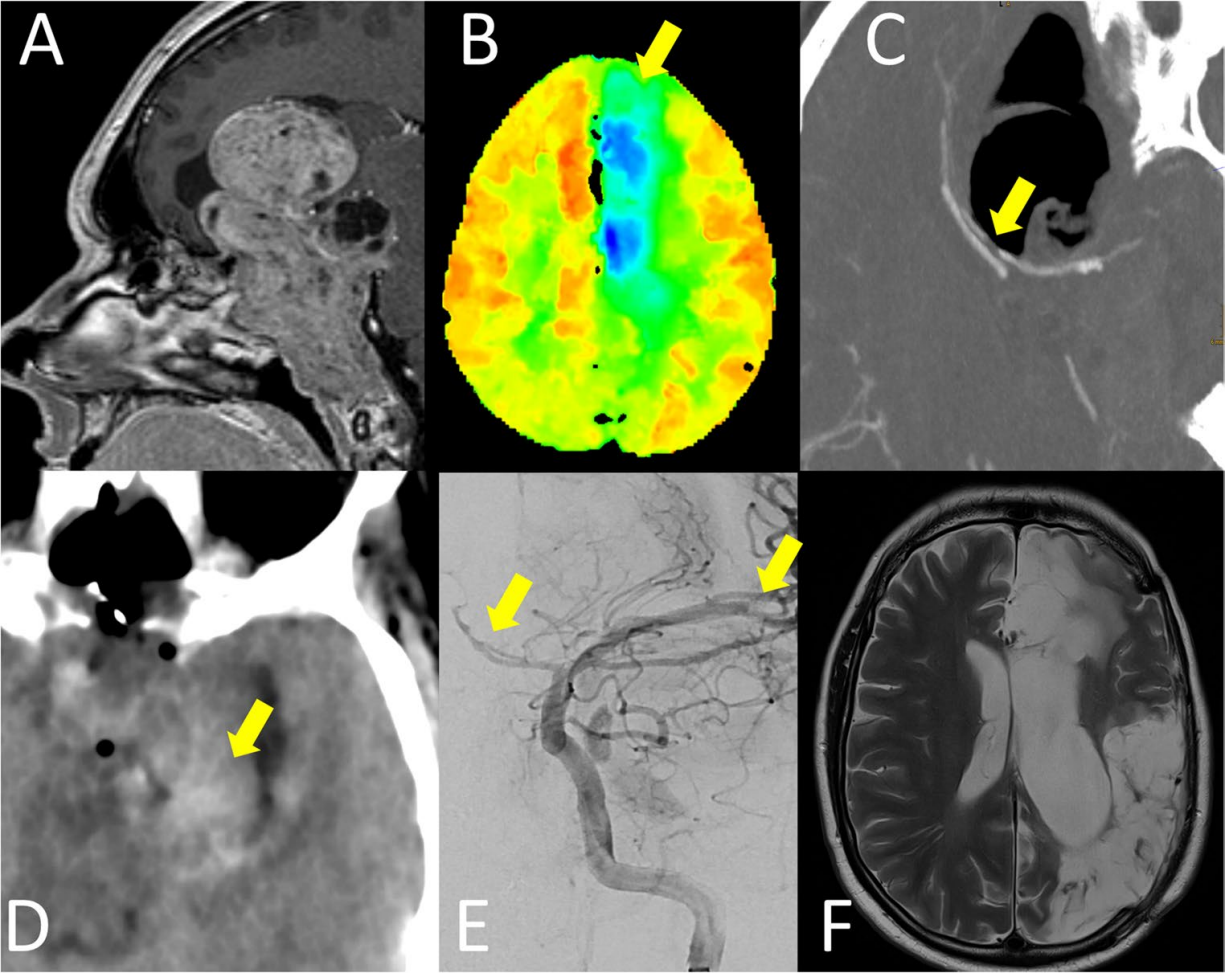
Case 3–45 year old male with giant silent corticotrope pituitary neuroendocrine tumor. **A**: preoperative sagittal contrast enhanced T1-weighted MR section showing extensive tumor infiltration of the anterior and middle skull base and clivus as well as a large suprasellar extension into the frontal lobe and third ventricle. **B**: Tmax map of perfusion CT on first postoperative day, blue areas indicating delayed perfusion, green yellow and red areas indicating timely perfusion, arrow pointing out perfusion deficit in the area supplied by the left anterior cerebral artery. **C**: Sagittal Reconstruction of contrast-enhanced CT angiography on postoperative day 1, arrowhead indicating focal interruption of left pericallosal artery. **D**: axial CT without contrast agent on postoperative day 1, arrow marking tumor bed hemorrhage predominantly on the left side. **E**: digital-subtraction angiography of the left internal carotid artery with arrows indicating reduced caliber of the anterior cerebral artery and interruption of the middle cerebral artery as signs of vasospasm. **F**: T2-weighted axial MR section demonstrating ischemic infarction of left anterior and middle cerebral artery territory

A 45 year old male (Fig. 4) presented with a slowly progressive loss of visual acuity especially on the left eye. At time of presentation he was only able to perceive light on the left eye. MR imaging demonstrated an extensive skull base lesion with infiltration of sella, sphenoid bone, cavernous sinus and clivus down to the jugular foramen on both sides as well a large suprasellar extension into the anterior and middle fossa with displacement of the left frontal and temporal lobe as well as the third ventricle. After a transsphenoidal biopsy led to histological confirmation of clinically silent corticotroph pituitary neuroendocrine tumor the patient was planned for surgical resection via a combined but successive transsphenoidal and transcranial approach in one session. Intraoperatively, an extensive resection of the extracavernous tumor was possible via the transsphenoidal route alone due to the soft consistency of the tumor, which is why the transcranial procedure was postponed. Most vessels of the anterior circulation up to the basilar artery and left mesial temporal lobe as well as the left optic and oculomotor nerves were skeletonized. Postoperatively, the patient had to be reintubated on the day of surgery due to low level of consciousness and a dilated left pupil. Emergent CT imaging showed a massive pneumocephalus and tumor bed hemorrhage without infarcts or perfusion deficit. One day later the patient was extubated but developed a right leg palsy. Repeated CT imaging now showed vasospasm of the left anterior and middle cerebral artery with associated perfusion deficit. Simultaneously the patient developed a cerebrospinal fluid leakage as well as arginine vasopressin deficiency and complete pituitary insufficiency. Both perfusion deficit and palsy regressed under hemodynamic augmentation and continuous intraarterial nimodipine therapy, which was started on the same day with low-molecular-weight heparin anticoagulation as is common practice in our department [36, 40, 58, 59]. On the fourth postoperative day the patient developed acute right hemiparesis and aphasia due to thrombosis in the M2 segment of the left MCA. After unsuccessful thrombectomy, a large MCA infarct developed with corresponding intracranial hypertension which led to decompressive hemicraniectomy. CSF leakage was controlled with external ventricular drainage, lumbar drainage and a secondary transsphenoidal reconstruction of the skull base after the patient had stabilized on the 18th postoperative day. After a long stay at the intensive care unit the patient was transferred to a rehabilitation center and received a cranioplasty but retained his predominantly expressive aphasia and hemiparesis at follow up after eight months. The early occurrence of vasospasm in this case suggests direct (dissection along the vessels) or indirect (mass shift) mechanical manipulation as causative.

## Systematic review

### Epidemiological data of reported cases

**Fig. 5:**
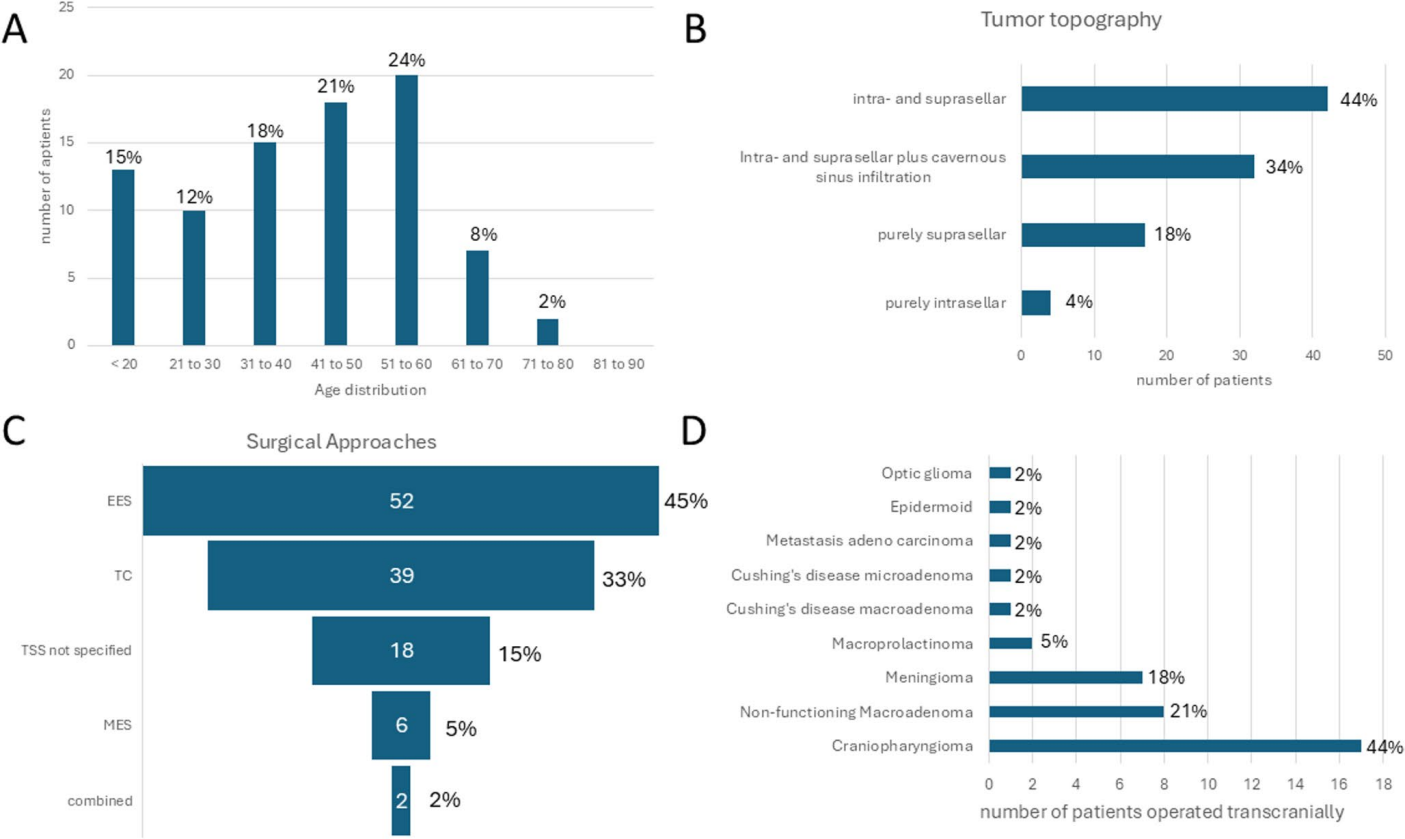
Epidemiological data of 114 in the literature reported cases and the three cases reported in this review. **A**: age distribution. **B**: tumor topography, most reported patients suffered from lesions with intra- and suprasellar extension, frequently with cavernous sinus infiltration. **C**: distribution of surgical approaches demonstrating that endonasal surgery was the most frequent approach. EES: endoscopic endonasal surgery, MES: microscopic endonasal surgery, TSS not specified: transsphenoidal approach without further specification, TC: transcranial approach. **D**: Entities operated on transcranially, most frequent reasons for a transcranial approach were craniopharyngioma, meningioma and non-functioning pituitary macroadenoma

**Fig. 6:**
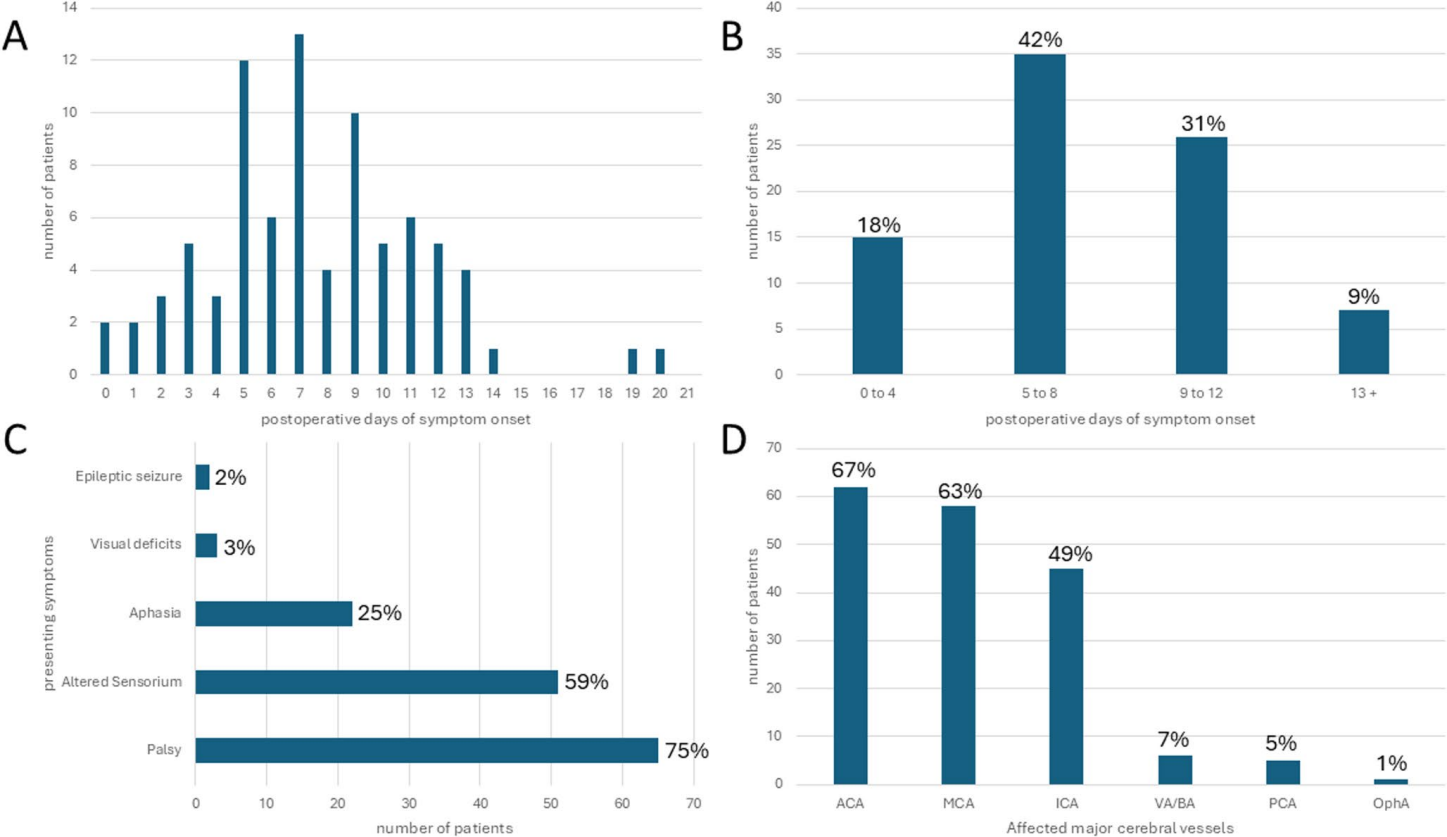
Distribution of reported vasospasm. **A**: Postoperative day of symptom onset by day. **B**: Postoperative day of symptom onset grouped from day 1–4, 5–8, 9–12 and afterwards, both diagrams demonstrate a distribution pattern similar to vasospasm/delayed cerebral ischemia in the context of aneurysmal subarachnoid hemorrhage. **C**: Symptoms at onset leading to diagnosis of vasospasm. **D**: Affected vessels as diagnosed by transcranial doppler sonography, CT or MR angiography or digital subtraction angiography after symptom onset. Anterior and middle cerebral artery were most frequently affected correlating with the frequent clinical presentation with palsy, altered sensorium or aphasia. ACA: anterior cerebral artery, MCA: middle cerebral artery, ICA: internal carotid artery, VA/BA: vertebral artery/basilar artery, PCA: posterior cerebral artery, OphA: ophthalmic artery

Overall and including the three original cases reported herein, 117 cases were extracted from 59 articles and reports [1–4, 6, 8–14, 16–21, 24, 27–31, 33–35, 37–39, 41, 42, 45, 48–53, 56, 57, 60–62, 64–72, 75, 77, 78, 80]. Among cases with reported sex (85/117, 72.6%), there was no predominance regarding one sex (41/85 or 48.2% male, 44/85 or 51.8% female). The age distribution reported in 85 patients is shown in Fig. 5 and peaks at the fifth and sixth decade followed by a sharp decline. Pathology was reported in 110/117 cases (94.0%). The most frequent pathological entities were non-functioning pituitary neuroendocrine tumors (50/117), craniopharyngioma (25/117) and perisellar skull base meningiomas (10/117). All pathological entities are listed in Table 1. Specific growth extension was reported or visually depicted in 95/117 cases (81.2%). Most lesions presented with either intra- and suprasellar extension or additional cavernous sinus extension (74/95, 77.9%). Highlighting that vasospasm is most frequently reported after surgery for lesions that extend beyond the sella (Fig. 6).

**Table 1:** Pathology summary

Disease	Number of Patients (%)
Non-functioning Pituitary Adenoma	50 (42.7%)
Craniopharyngioma	25 (21.4%)
Meningioma	10 (8.4%)
Prolactinoma	6 (5.1%)
Acromegaly	4 (3.4%)
Cushing’s Disease	4 (3.4%)
Rathke’s Cleft Cyst	2 (1.7%)
Atypical Adenoma	2 (1.7%)
Gonadotrope Adenoma	2 (1.7%)
Epidermoid	1 (0.9%)
Optic glioma	1 (0.9%)
Metastasis of Neuroendocrine Tumor	1 (0.9%)
Lymphocytic Hypophysitis	1 (0.9%)
Metastasis of Adenocarcinoma	1 (0.9%)
Not Specified	7 (6.0%)
Total	117 (100%)

The operative approach was reported in all cases. While 76/117 cases (65.0%) were operated via the transsphenoidal route (52 endoscopically, 6 microsurgically, 17 not specified), 39/117 (33.3%) were approached transcranially and two cases combined transsphenoidally and transcranially. Most of the transsphenoidal cases that were not specified date to the pre-endoscopic era and thus were probably operated on microsurgically. The most common indication for a transcranial approach was craniopharyngioma (17/39, 43.6%, Fig. 5). Out of 73 cases with reported extent of resection (62.4%), total resection was achieved in 45 (61.6%), whereas subtotal resection was reported in 28 cases (38.4%).

### Characteristics of reported vasospasm

Case-specific onset of symptomatology was reported in 83/117 cases (70.9%). The most frequent onset was between the fifth and twelfth postoperative day (61/83, 73.5%). In twelve cases the day of onset was not specifically reported, whereas two case series of 6 and 16 cases reported only a group average postoperative day of onset at fourth and third day respectively (Fig. 5). Symptomatology was described in 87/117 cases (74.4%, Fig. 6). The large majority of patients presented with new onset of palsy (65/87, 74.7%), altered sensorium (51/87, 58.6%) or aphasia (22/87, 25.3%). Correspondingly, vasospasm affected – almost as a rule – the major vessels of the anterior circulation: internal carotid artery (ICA) or anterior cerebral artery (ACA) or middle cerebral artery (MCA) and their various combinations represent 93.2% of reported affected vessels in 92/117 cases (78.6%). The posterior circulation, on the other hand, represented only 6.2% of affected cerebral arteries (Fig. 6). Of note, among 67/117 (57.3%) cases for which postoperative CT or MR imaging was shown or described, 50 (74.6%) were associated with postoperative tumor bed hemorrhage or subarachnoid hemorrhage (Table 2). Arachnoid violation could be assessed in 98/117 cases (83.8%): among these it was reported in 75.5% (73/98).

**Table 2:** Extent of resection, reported frequency of tumor bed hemorrhage, arachnoid violation, postoperative electrolyte disturbance and vasospasm-related infarction on last imaging. Of note, while all percentages in the text refer to the number of cases that actively reported each variable, percentages in this table refer to the absolute number of cases included in this review, since the amount of cases not reporting the variables listed above are also specified

	Number of patients (%)
Extent of resection	
Total	45 (38.5%)
Subtotal	28 (23.9%)
Not specified	44 (37.6%)
Tumorbed hemorrhage	
Yes	50 (42.7%)
No	17 (14.6%)
Not specified	50 (42.7%)
Arachnoid violation	
Yes	73 (62.4%)
No	25 (21.4%)
Not specified	19 (16.2%)
Postoperative electrolyte disturbance	
Yes	47 (40.2%)
No	8 (6.8%)
Not specified	62 (53.0%)
Infarction on last imaging after vasospasm	
Yes	50 (42.7%)
No	15 (12.8%)
Not specified	52 (44.5%)
Total	117 (100%)

Of note, two cases were reported concurrent with postoperative formation of a pseudoaneurysm of the ICA, while eight cases were reported in association with postoperative meningitis.

Neurological outcome was reported in 98/117 cases (83.8%) with a return to neurological baseline in 43/98 (43.9%), whereas neurological morbidity and mortality were recorded in 36.7% and 19.4%, respectively (36/98 and 19/98 cases). In 65/117 cases with cerebral imaging at the end of treatment 50/65 (76.9%) presented infarction (Table 2). Postoperative electrolyte status was only reported in less than half of patients (56/117, 47.9%), of which 85.5% developed transient or permanent postoperative electrolyte disturbance presenting as either arginine vasopressin deficiency (40/56, 71.4%) and/or hyponatremia (24/52, 46.2%).

The reported therapeutic regimes varied depending on decade, institutional protocols or standard operating procedures and comprised variations of oral and intravenous nimodipine, steroids, triple H-therapy (hemodilution, hypertension, hypervolemia), hemodynamic augmentation, intraarterial applications of nimodipine, nifedipine, nicardipine, verapamil, fasudil, milrinone, papaverine and balloon or stent retriever angioplasty. However, reliable analysis of therapeutic regimes was not possible due to a high variability in reported terminology and degree of detail.

In summary, postoperative vasospasm after pituitary surgery is most often reported after transsphenoidal resection of large non-functioning pituitary adenomas or transcranial surgery of craniopharyngiomas. Vasospasm manifests predominantly between the fifth and twelfth postoperative day with palsy, altered sensorium or aphasia and affects ICA, MCA, ACA or their combinations in more than 90% of cases. Therapy is not standardized, while more than half of reported patients suffer permanent neurological morbidity (36.7%) or mortality (19.4%). Tumor bed hemorrhage and arachnoid violation were present in 74.6% and 75.5% of assessable cases, respectively.

## Discussion

The inherent bias of this review of literature on cerebral vasospasm after transsphenoidal or transcranial pituitary surgery is that vascular imaging is not routinely performed after pituitary surgery. Thus, vasospasm is commonly detected and reported only after an unusual postoperative course that is not attributed to hormonal or electrolyte disturbances, CSF leakage, direct manipulation of the optic apparatus or postoperative meningitis. This is highlighted by the fact that all reviewed cases were detected due to unexplained new neurological deficits after surgery (although the MeSH-terms did not filter exclusively symptomatic vasospasm). This might misrepresent the danger of postoperative vasospasm in pituitary surgery especially since it is known from subarachnoid hemorrhage that only severe vasospasm causes a hemodynamically relevant perfusion deficit [25, 73]. While this review surveys a large part of the literature on this subject in existence, this selection and detection bias severely limits the interpretability of the source data.

Additionally, while sellar and suprasellar lesions naturally show a topographical proximity especially to the multitude of small perforating arteries of the infundibulum, optic apparatus, hypothalamus, anterior and posterior perforated substance, vasospasm of these vessels and small ischemic infarcts in their respective territories are difficult and often impossible to visualize with conventional cranial imaging. These circumstances may explain why this review exclusively found vasospasm of large cerebral vessels in the context of new postoperative neurological deficits (with one case of vasospasm of the ophthalmic artery).

Nevertheless, while several recent reviews have summarized only a very limited amount of 12, 30, 14, 41 and 60 (general craniotomy with a subset of pituitary) cases respectively, this review is the most comprehensive on this topic to date and demonstrates that postoperative vasospasm constitutes a pertinent issue in transsphenoidal and transcranial surgery of the pituitary and its surroundings as many sellar and suprasellar tumors show a close topographical relationship to the circle of Willis and an increasing number of cases are reported [5, 20, 24, 61, 72]. The reported numbers of this report diverge from others due to several reasons: firstly, this report does not focus exclusively on pituitary adenomas but also comprises Rathke’s cleft cysts, craniopharyngiomas, tuberculum sellae meningiomas and rarer entities. Secondly, inclusion was performed independently from the type of approach and patient age. Thirdly, meticulous examination not only of the Pubmed-database but also all bibliographies of all included articles enabled inclusion of several cases that were not listed on Pubmed. Furthermore, the inclusion of abstracts that were found via the keyword ‘delayed neurological deficit’ and consequently checked for evidence of vasospasm allowed including cases in which vasospasm was not primarily highlighted in title or keywords. Lastly, cases from larger surgical series reporting single cases of vasospasm en passant were also included. However, because only the Pubmed-database was formally queried, there is no claim for completeness as regards world literature.

Vasospasm has the potential of life threatening neurological sequelae demonstrated by the fact that more than half of patients reported in the literature suffer neurological morbidity or mortality. The potential pathophysiological mechanisms classically cited in those reports are (1) direct mechanical manipulation and damage to the vessel wall, (2) release of chemical substances during tumor removal, (3) vasoconstrictive properties of blood and its breakdown products in the basal cisterns after surgery and (4) hypothalamic damage. This case collection demonstrates a similar distribution of delayed vasospasm (delayed onset in 73.5% of cases specified from 5th to 12th postoperative day) as has been known from spontaneous subarachnoid hemorrhage [25, 73], which is strongly associated with the amount of subarachnoid blood [22, 26, 54, 55]. This is further supported by the fact that, although not systematically reported, in 74.6% of cases in which early postoperative imaging was shown or described tumor bed hemorrhage in surgery of large tumors of the sella naturally occurring in the subarachnoid space in the basal cisterns was reported. Correspondingly, arachnoid violation occurred in 75.5% of assessable cases. While there are no systematic studies regarding the prevalence of postoperative tumor bed hemorrhage in asymptomatic patients after pituitary surgery similar to asymptomatic postoperative mild or moderate vasospasm, this is in line with the fact that postoperative vasospasm in the reported cases almost exclusively affects the vessels at the operative site (ICA, ACA, MCA in 93.2%) which are most likely to be affected by tumor bed hemorrhage, because it is usually not as diffuse as spontaneous aneurysmatic subarachnoid hemorrhage. Fisher originally linked especially thick clots, rather than diffuse subarachnoid blood, to a higher risk of vasospasm. Similar to Fisher’s findings, vasospasm appears more common in the anterior cerebral artery than in the middle cerebral artery [26]. This implies an association between tumor bed hemorrhage, arachnoid violation and vasospasm of the anterior circulation. Of note, while this review reports a lower mortality rate of 19.4% compared to 38% reported by Tarimah et al. and 22.5% reported by Doat-Sarfati et al., those three reviews combined stress the danger that emenates from this rare postoperative complication, necessitating a low threshold for vascular diagnostics and treatment in case of detected vasospasm [20, 72].

Nonetheless, 15 cases (18.1% of cases specified) with a reported onset within the first four postoperative days also suggest the possibility of a direct mechanical origin either due to direct manipulation or strain due to rapid vessel shift after resection of large masses in a short period of time since most tumors associated with reported postoperative vasospasm showed supra- or parasellar extension (77.9% of specified cases). On the one hand, involvement of basal cerebral arteries by suprasellar craniopharyngiomas is a well known risk and causes many surgeons to choose a transcranial approach for better control of the vasculature. This is confirmed in this review since the most common indication for a transcranial approach was craniopharyngioma resection (17 out of 36 transcranial approaches). On the other hand, by far the most frequent entity reported in association with postoperative vasospasm were non-functioning pituitary adenomas (50/117, 42.7%). As these lesions are often only discovered due to their mass effect they may develop a considerable size with involvement and even infiltration of the basal cerebral arteries as well as a high risk for postoperative hemorrhage. Thus, diagnostic threshold should be particularly low after resection of large non-functioning pituitary adenomas.

Considering the marked similarity of the characteristics reported above with vasospasm after spontaneous subarachnoid hemorrhage, application of well established treatment algorithms should be evaluated. This comprises firstly identification of patients at risk, especially with large and giant-sized non-functioning pituitary adenomas, suprasellar subarachnoid craniopharyngiomas, brisk intraoperative bleeding or postoperative imaging with tumor bed hemorrhage and direct vessel dissection. Secondly, patients at risk might benefit from intraoperative irrigation and prophylactic treatment with nimodipine, especially if the high rates of morbidity and mortality are taken into account. While no difference in outcome could systematically be shown, in our department placement of a central line and continuous intravenous application is preferred due to higher serum concentrations and more reliable application in patients that suffer from acute neurological disease. Thirdly, there should be a low threshold for imaging such as CT angiography and perfusion in patients at risk to detect vasospasm or delayed ischemia. Considering the susceptibility of the anterior cerebral arteries and their hypothalamic perforators, behavioral abnormalities and personality changes, disorientation and altered sensorium should be screened for particularly in addition to paresis and aphasia.

Nevertheless, this review indicates the limited knowledge and evidence on clinical characteristics of postoperative vasospasm in pituitary surgery which are based on case reports and small case series with inherent reporting bias. In order to systematically gather evidence on this phenomenon implementation of a screening protocol for both postoperative tumor bed hemorrhage and vasospasm of intracranial arteries – preferably as part of a systematic registry for sellar pathologies – is necessary. The incidence of asymptomatic tumor bed hemorrhage and asymptomatic postoperative vasospasm remains unclear.

### Limitations

Limitations of this review are the selection and detection bias inherent to a retrospective review of mostly single case reports and small series of a presumably rare complication. Both postoperative CT and MRI as well as vascular imaging are not routinely performed after pituitary surgery in many centers. Thus, cases are more likely to be reported due to an unexpectedly bad clinical course, overrepresenting severe vasospasm. Similarly, the frequency of asymptomatic postoperative tumor bed hemorrhage is not known. Inconsistency in reporting clinical data across many authors in eight decades leads to very inhomogenous source data, necessitating to specify the amount of cases that each area of interest was reported in and leading to a constantly changing reference population. Particularly reports on therapeutic details and success was fragmentary impeding a coherent report on this topic. Lastly, this review queried only Pubmed-database and all bibliographies of included articles, thus not reviewing the entirety of world literature. In summary, this review is limited to reporting biased associations, while there is no ground for causal inference.

## Conclusion

Postoperative vasospasm after pituitary surgery is most often reported after transsphenoidal resection of large non-functioning pituitary adenomas or transcranial surgery of craniopharyngiomas. Vasospasm manifests predominantly between the fifth and twelfth postoperative day with altered sensorium, palsy or aphasia and affects ICA, MCA, ACA or their combinations in more than 90% of cases. Therapy is not standardized, while more than half of reported patients suffer permanent neurological morbidity (37.6%) or mortality (19.4%). In order to systematically gather evidence on this phenomenon implementation of a screening protocol for both postoperative tumor bed hemorrhage and vasospasm of intracranial arteries – preferably as part of a systematic registry for sellar pathologies – is necessary.

## Data Availability

No datasets were generated or analysed during the current study.
